# Effect of Nd:YAG and Diode Lasers on Apical Seal of Root Canals Filled with AH Plus and Mineral Trioxide Aggregate-Based Sealers

**Published:** 2018-01

**Authors:** Elham Khoshbin, Zakiyeh Donyavi, Erfan Abbasi Atibeh, Shahin Kasraei, Rasoul Yousefimashouf, Ghodratollah Roshanaei, Faranak Amani

**Affiliations:** 1 Assistant Professor, Department of Endodontics, School of Dentistry, Hamadan University of Medical Sciences, Hamadan, Iran; 2 Assistant Professor, Department of Periodontics, School of Dentistry, Alborz University of Medical Sciences, Karaj, Iran; 3 Professor, Department of Restorative Dentistry, School of Dentistry, Shahid Beheshti University of Medical Sciences, Tehran, Iran; 4 Associate Professor, Department of Microbiology, Hamadan University of Medical Sciences, Hamadan, Iran; 5 Associate Professor, Modeling of Noncommunicable Diseases Research Center, Department of Biostatistics and Epidemiology, School of Public Health, Hamadan University of Medical Sciences, Hamadan, Iran; 6 Assistant Professor, Department of Endodontics, School of Dentistry, Alborz University of Medical Sciences, Karaj, Iran

**Keywords:** Lasers, Endodontic Sealer, Root Canal Obturation

## Abstract

**Objectives:**

Laser irradiation, as an adjunct to root canal preparation, may increase the success rate of endodontic treatments. This study aimed to assess the effect of neodymium-doped yttrium aluminum garnet (Nd:YAG) and diode lasers on the apical seal of the root canals filled with AH Plus® and mineral trioxide aggregate (MTA)-based sealers.

**Materials and Methods:**

This in-vitro experimental study was conducted on 96 single-rooted, single-canal extracted human teeth with closed apices. The root canals were prepared by using ProTaper® rotary instruments and were randomly divided into six groups (n=16): 940-nm diode laser and AH Plus® sealer (group 1), Nd:YAG laser and AH Plus® sealer (group 2), AH Plus® sealer (group 3), 940-nm diode laser and MTA-based sealer (group 4), Nd:YAG laser and MTA-based sealer (group 5), MTA-based sealer (group 6), as well as positive and negative control groups. A bacterial leakage model was used for microleakage assessment. Qualitative assessment was done by using a scanning electron microscope (SEM). Data were analyzed by two-way analysis of variance (ANOVA) at the significance level of 0.05.

**Results:**

There were statistically significant differences between the experimental and control groups (P=0.002). The laser-treated groups showed a lower apical microleakage compared to the non-laser-treated groups, although the difference was not statistically significant (P>0.05). No significant differences were noted between the two lasers in terms of the apical microleakage, irrespective of the type of sealer (P>0.05).

**Conclusions:**

Laser irradiation, as an adjunct to root canal preparation, has no significant effect on the level of apical microleakage.

## INTRODUCTION

The success of an endodontic treatment depends on a number of factors including a correct diagnosis and treatment planning, adequate cleaning and shaping of the root canal system, and an efficient three-dimensional (3D) root canal obturation [1]. An efficient obturation of the root canal system can be achieved through complete chemomechanical preparation of the root canal system, which plays an important role in the prevention of bacterial leakage and recontamination of the root canal system [2,3]. Elimination of necrotic pulp tissues, residual microorganisms, dentinal chips and debris, and the smear layer produced during endodontic instrumentation is a prerequisite for a successful endodontic treatment [4]. Several studies have shown that root canal preparation methods result in the production of a smear layer, which adheres to dentinal walls and allows for lodging of bacteria. The smear layer also limits the access of irrigating solutions and intracanal medicaments to dentinal tubules.

Therefore, elimination of the smear layer enhances the sealing and resistance against bacterial penetration [3,4]. Several studies have recommended the application of sodium hypochlorite (NaClO) to remove the organic components of the smear layer, and ethylenediaminetetraacetic acid (EDTA) disodium to remove the inorganic components of the smear layer in order to obtain a cleaner dentinal surface; however, these methods do not completely eliminate the smear layer [2,5]. Evidence shows that root canal preparation followed by laser irradiation, as an adjunct, may yield a higher success rate [6,7]. Lasers are valuable tools in endodontic treatment, and it appears that different laser wavelengths can decrease the population of growing microorganisms in the root canal [6,7]. Laser irradiation also causes significant changes in the dentinal walls of root canals. Laser irradiation can affect dentin permeability by melting the dentin and obstructing the dentinal tubules [1]. Evidence shows that the neodymium-doped yttrium aluminum garnet (Nd:YAG) laser is absorbed by mineral structures such as hydroxyapatite carbonate and phosphate [3]. The Nd:YAG laser destructs the crystalline structures through its thermomechanical action and causes morphological changes in the dentin by melting and resolidification. The laser enhances root canal sealing and decreases dentin permeability by deposition of glass-like substances [3]. Diode laser is a relatively new tool, which has gained increasing popularity in endodontics due to its small size and flexible thin fibers, making it suitable for use in curved root canals. Evidence shows that a diode laser with a 980-nm wavelength eliminates the bacteria deeply lodged in dentinal tubules. Also, in the process of dentin melting, the diode laser creates a more uniform circular pattern compared to that created by the Nd:YAG laser. However, the effects of diode laser on apical microleakage and dentin morphology have not been well-studied [8,9]. Standard obturation of the root canal system with gutta-percha and endodontic sealers is performed for 3D filling of the root canal system and prevention of reentry of microorganisms [1].

Several sealers with different properties are available in the market. AH Plus® is a commonly used sealer in endodontic treatments, which has optimal sealing properties and is used as the gold standard with the ability to bond to the radicular dentin [2]. Mineral trioxide aggregate (MTA)-based sealers are also increasingly used. These sealers have optimal biological and physical properties [10]. They also induce hard tissue conductivity and are bioactive and biocompatible [11].

A search of the literature yielded no previous study on the effects of different lasers on the apical microleakage of the root canals filled with the mentioned sealers. Moreover, studies on the comparison of the efficacy of Nd:YAG and diode lasers are limited, and the existing ones have reported controversial results [8,9,12]. Also, most previous studies on microleakage have used dye penetration technique, whereas microleakage assessment by a bacterial leakage model and scanning electron microscopy (SEM) is more accurate and better simulates the clinical setting [13]. Therefore, this study aimed to assess and compare the effects of Nd:YAG and diode lasers on the apical microleakage of the root canals filled with AH Plus® and MTA-based sealers using a bacterial leakage model. The null hypothesis was that the two lasers would have no significant effect on the level of apical microleakage in the root canals filled with the mentioned sealers.

## MATERIALS AND METHODS

This in-vitro experimental study was conducted on 96 single-rooted, single-canal human teeth with mature apices. The study protocol was approved by the ethics committee of Hamadan University of Medical Sciences (Ethical code: 195). The sample size was calculated to be 32 teeth in each group according to a previous study by Moura-Netto et al [7], and by considering a study power of 90% and type I error of 5%. Thus, a total of 96 teeth were included in the study.

The inclusion criteria for the teeth consisted of having a single canal with a closed apex, no calcification, no internal or external root resorption, no curvature and no other anomaly (confirmed by taking periapical radiographs). The teeth were disinfected in 5.25% NaClO solution for two hours and were then stored in distilled water until the experiment [10]. The crowns were cut at 2mm above the cementoenamel junction (CEJ) [10]. The teeth with root lengths of 12±0.5mm after cutting the crowns were chosen for the study. A K-file #10 (MANI Inc., Utsunomiya, Japan) was inserted into the root canal until its tip was visible at the apex. The working length (WL) was determined 1mm short of this length [9]. The root canals were prepared by using ProTaper® rotary files (Dentsply Maillefer, Ballaigues, Switzerland) operated at 300 rpm as recommended by the manufacturer and according to the crown down technique. First, the coronal third was prepared by using the SX file. The S1 file was used for canal instrumentation 4mm short of the WL. The S1 and S2 files were used to the WL. Afterwards, the F1, F2 and F3 files were used to the WL [14]. The smear layer was removed using 5ml of 17% ethylenediaminetetraacetic acid solution (EDTA, ApadanaTak Co., Tehran, Iran) for five minutes and 5ml of 5.25% NaClO solution (Domestos, Unilever, Qazvin, Iran) for five minutes using a 30-gauge needle [10]. A final rinse with distilled water was then carried out [9]. To standardize the size of the apical foramen and to prevent errors due to different sizes of the foramen, a K-file #45 was passed through the apex by 2mm [8]. The teeth were then randomly divided into six groups (n=16) as follows:

Group 1: Irradiation of a 940-nm diode laser (Epic10, Biolase Inc., Irvine, Canada) and obturation with the AH Plus® sealer (Dentsply, Konstanz, Germany)
Group 2: Irradiation of a 1064-nm Nd:YAG laser (Fidelis Plus III, Fotona, Ljubljana, Slovenia) and obturation with the AH Plus® sealer
Group 3: No laser irradiation, obturation with the AH Plus® sealer
Group 4: Irradiation of the 940-nm diode laser and obturation with the MTA-based sealer (Angelus, Londrina, PR, Brazil).
Group 5: Irradiation of the Nd:YAG laser and obturation with the MTA-based sealer
Group 6: No laser irradiation, obturation with the MTA-based sealer

Sixteen teeth were used as positive and negative controls [3].

Positive control group: No laser irradiation, obturation with gutta-percha without sealer. Negative control group: No root canal preparation and no obturation were performed. Root surfaces including the apical foramen and the root canal’s orifice were covered with two layers of nail varnish (Bourjois, Paris, France) [2].

In the groups irradiated with the 940-nm diode laser, 300-μm fibers with a 0.1-W output power in the continuous mode were used to irradiate the root canal walls in a circular fashion at a speed of 2mm/second. This was repeated four times at 20-second intervals [7]. In the groups irradiated with the 1064-nm Nd:YAG laser, 300-μm fibers with an output power of 2W, the energy density of 50 mJ/second and frequency of 15Hz were used to irradiate the root canal walls in a circular fashion at a speed of 2mm/second according to the helicoidal technique used by Gutknecht et al; this was repeated four times at 20-second intervals [8]. AH Plus® and MTA-based sealers were prepared according to the manufacturers’ instructions. The master cone was dipped in the sealer and was inserted into the root canal. The root canals were filled according to the cold lateral compaction technique by using a finger spreader by a single operator. After obturation, excess gutta-percha was cut at the orifice, and the remaining gutta-percha was condensed [8]. The coronal third of the root canal was sealed with a glass-ionomer cement. The teeth were then incubated at 37°C and 100% moisture for 48 hours.

### Microbial leakage analysis:

External root surfaces were covered with two layers of nail varnish, except for 2mm around the apex [10]. The positive control teeth were obturated without using a sealer as described above, and the root surfaces were covered with nail varnish, except for 2mm around the apex. The negative control teeth remained unfilled, and the external root surfaces including the apex were covered with nail varnish [15]. The teeth were placed in cut-end microtubes. Then, this assembly and glass vials were autoclaved at 121°C under a 2.1-bar pressure for 15 minutes. The microtubes containing the teeth were placed in the rubber caps of the glass vials and were transferred to larger vials containing 10ml of tryptic soy broth culture medium (Merck KGaA, Darmstadt, Germany) under aseptic conditions. The tryptic soy broth culture medium holding 1.5×108 colony forming units (CFU)/ml of Enterococcus faecalis (E.faecalis) bacteria in a total volume of 1ml was injected into the upper compartment of the assembly by a 27-gauge sterile needle. The microleakage assessment was done by observing the turbidity of the medium [16]. The samples were monitored for turbidity for 90 days (for obtaining statistically valid results and by considering that the materials’ aging may change the ecological micro-environment), and injection of the bacterial suspension was performed every three days. In case of occurrence of turbidity, a sample of the medium was cultured in blood agar (Merck KGaA, Darmstadt, Germany) to ensure that the turbidity was caused by E.faecalis [10]; upon confirmation, the date of observation of turbidity was noted.

### SEM assessment:

Two teeth of each group were selected for qualitative assessment under an SEM (JSM-810A, JEOL Ltd., Tokyo, Japan). Superficial longitudinal grooves were created on the buccal and lingual surfaces of the roots by using a diamond disc. Using these grooves, the roots were fractured by a chisel and a surgical hammer. Another groove was created at 3mm above the apex, and the root was fractured again to obtain a sample of the root for SEM assessment. The specimens were gold-palladium sputter coated (JEOL Ltd., Tokyo, Japan) and were evaluated under the SEM at ×25, ×300, and ×700 magnifications operating at 15 kilovoltages (kV) with a 25-mm working distance to assess the dentinal tubule openings, the presence of a smear layer, and adaptation and penetration of the sealer [3].

### Statistical analysis:

Data were analyzed using SPSS 16 software program (IBM Co., Chicago, IL, USA). The groups were compared using two-way analysis of variance (ANOVA). P<0.05 was considered statistically significant.

## RESULTS

### Microbial apical microleakage:

**Fig. 1:**
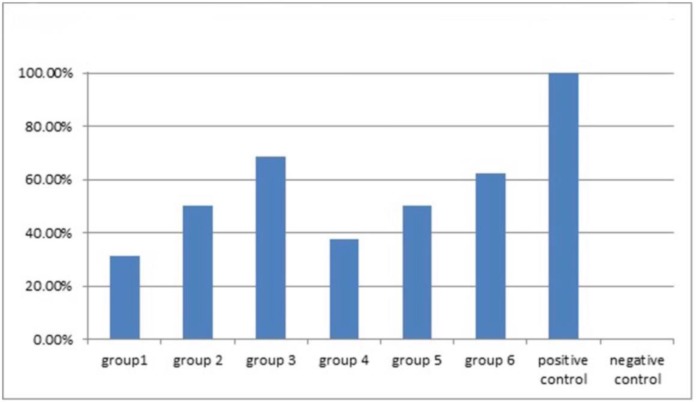
The percentage of apical bacterial microleakage incidence in study groups over 90 days

All the teeth in the positive control group and none of the teeth in the negative control group showed bacterial microleakage. The incidence of apical microleakage was 31.5% in group 1, 50% in group 2, 68.75% in group 3, 37.5% in group 4, 50% in group 5, and 62.5% in group 6. Group 1 (diode laser and AH Plus® sealer) showed the lowest, and group 3 (no laser, AH Plus® sealer) showed the highest incidence of apical microleakage (Fig. 1). Two-way ANOVA was used for the evaluation of the effect of lasers and sealers on the apical microleakage, and the results were not significantly different (P>0.05).

Table 1 shows the mean time of occurrence of microleakage in the groups. As shown in this table, the shortest mean time of occurrence of apical microleakage was 6.31 days in group 4, and the longest period was 19.19 days in group 3. According to the Kruskal-Wallis test, the difference in this regard was not significant among the groups (P=0.066).

**Table 1.** Mean time of occurrence of apical microleakage in the studied groups (Kruskal-Wallis test)

**Group**	**Number of samples**	**Mean time period (days)**	**Standard deviation**	**P-value**
1	16	10.00	22.32	0.066
2	16	10.19	17.31	
3	16	19.19	24.90	
4	16	6.31	11.44	
5	16	16.00	27.77	
6	16	14.50	22.27	
Positive control	8	2.25	0.71	
Negative control	8	0.00	0.00	

### SEM analysis:

**Fig. 2:**
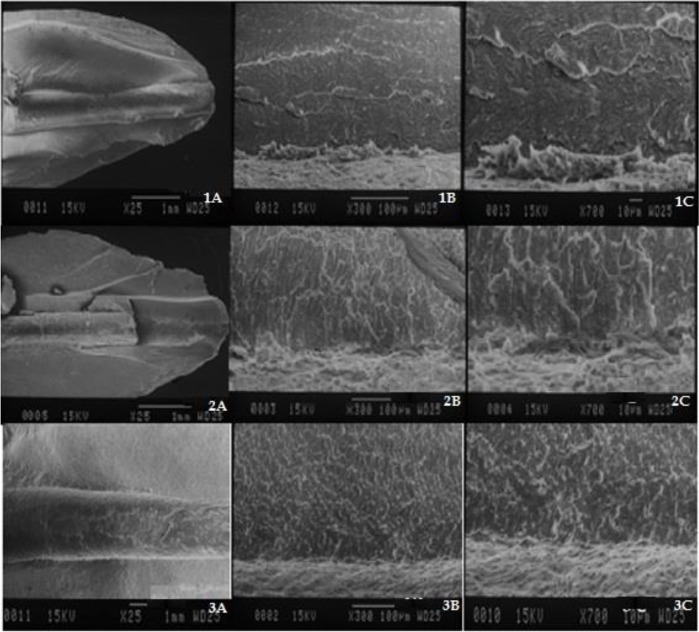
SEM micrographs of group 1 (diode laser and AH Plus® sealer). (1A) ×25 magnification. (1B) ×300 magnification. (1C) ×700 magnification. SEM micrographs of group 2 (Nd:YAG laser and AH Plus® sealer). (2A) ×25 magnification. (2B) ×300 magnification. (2C) ×700 magnification. SEM micrographs of group 3 (no laser, AH Plus® sealer). (3A) ×25 magnification. (3B) ×300 magnification. (3C) ×700 magnification

**Fig. 3:**
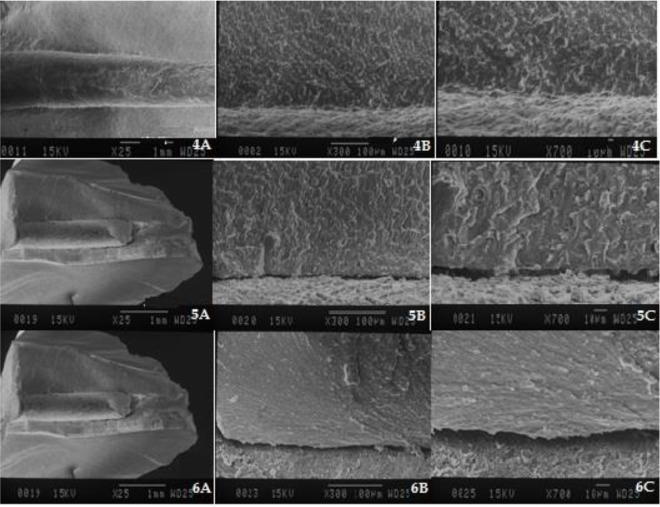
SEM micrographs of group 4 (diode laser, MTA-based sealer). (4A) ×25 magnification. (4B) ×300 magnification. (4C) ×700 magnification. SEM micrographs of group 5 (Nd:YAG laser, MTA-based sealer). (5A) ×25 magnification. (5B) ×300 magnification. (5C) ×700 magnification. SEM micrographs of group 6 (no laser, MTA-based sealer). (6A) ×25 magnification. (6B) ×300 magnification. (6C) ×700 magnification

**Fig. 4:**
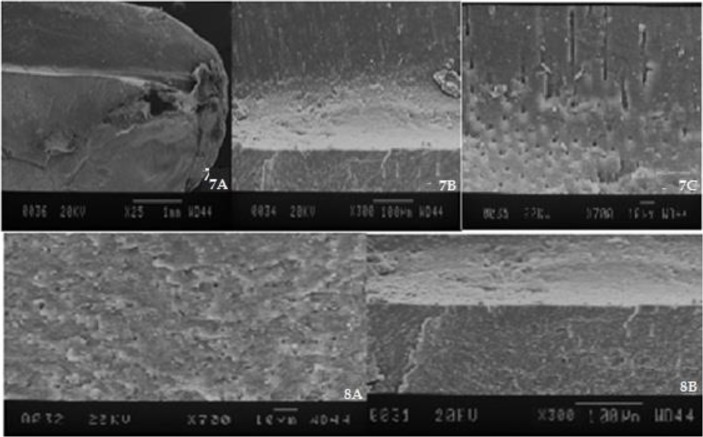
SEM micrographs of the negative control group. (7A) ×25 magnification. (7B) ×300 magnification. (7C) ×700 magnification. SEM micrographs of the positive control group. (8A) ×300 magnification. (8B) ×700 magnification

The followings were observed in each group:

*Group 1:* No gap between the root filling material and dentinal walls (an excellent adaptation; Fig. 2. 1A), relative obstruction of dentinal tubules’ orifices, adhesion of the sealer to dentinal walls, penetration of the sealer into dentinal tubules, the presence of a modified smear layer (Fig. 2. 1B, 1C).
*Group 2:* No gap between the root filling material and dentinal walls (an excellent adaptation; Fig. 2. 2A), complete obstruction of dentinal tubules’ orifices (a uniform surface), an optimal adaptation of the sealer to dentinal walls without penetration into or adhesion to dentinal tubules, no smear layer (Fig. 2. 2B, 2C).
*Group 3:* No gap between the root filling material and dentinal walls (an excellent adaptation; Fig. 2. 3A), open dentinal tubules, relative presence of the sealer, an optimal adaptation of the sealer to dentinal walls without penetration into dentinal tubules (Fig. 2. 3B, 3C).
*Group 4:* Gaps between the root filling material and dentinal walls (a poor adaptation, Fig. 3. 4A), relative obstruction of dentinal tubules without penetration of the sealer into the dentinal tubules, a poor adaptation of the sealer to dentinal walls (Fig. 3. 4B, 4C).
*Group 5:* Gaps between the root filling material and dentinal walls (a poor adaptation, Fig. 3. 5A), complete obstruction of dentinal tubules’ orifices, a poor adaptation of the sealer to dentinal tubules, no smear layer (Fig. 3. 5B, 5C).
*Group 6:* No gap between the root filling material and dentinal walls (an excellent adaptation; Fig. 3. 6A), open dentinal tubules, relative presence of a smear layer, a good adaptation of the sealer to dentinal walls, no penetration of the sealer into dentinal tubules (Fig. 3. 6B, 6C).
*Negative control group:* Completely open dentinal tubules, absence of a smear layer (Fig. 4. 7A–7C)
*Positive control group:* Completely open dentinal tubules, presence of a smear layer (Fig. 4. 8A, 8B)

## DISCUSSION

Complete 3D filling of the root canal system is an important step in the prevention of bacterial microleakage and reinfection of the root canal system [3]. Nd:YAG and diode lasers were used in our study, and their efficacies in the prevention of apical microleakage were compared [6]. The Nd:YAG laser affects dentin permeability, and thus, has been extensively studied [2]. Diode laser is a relatively new tool, suitable for use in curved root canals [8,9]. Apical microleakage has been evaluated by different techniques in previous studies such as optical coherence tomography (OCT) technology [1], dye penetration [3,8,9,17], silver nitrate penetration [7,14,18,19], bacterial leakage models [10], fluid filtration [12], SEM [12,14,20], and stereoscopy [20]. We used a bacterial leakage model for the assessment of apical microleakage since it better simulates the clinical setting and has a high accuracy [13]. Qualitative assessments were carried out by using SEM. Our results showed that the incidence of apical microleakage was lower when the MTA-based sealer was used compared to the use of the AH Plus® sealer, but the difference was not significant. In 2016, Asawaworarit et al [21] compared the sealing ability of MTA Fillapex and AH Plus® sealers in warm vertical condensation technique by fluid filtration test. They showed that one week after obturation, the AH Plus® provided a better apical seal than the MTA Fillapex sealer. However, after four weeks, the apical seal provided by the MTA Fillapex was significantly superior to that provided by the AH Plus® sealer. This difference can be attributed to the composition of the MTA Fillapex sealer and the continuous formation of hydration products over time, which initiate the formation of calcium phosphate precipitates [21]. Their results after four weeks were similar to our results after three months. However, Sonmez et al [22], Gandolfi and Prati [23], and Vasconcelos et al [24] found different results. They used different methods of microleakage assessment such as dye penetration test and fluid filtration method. The difference in the results may be due to the use of different techniques for microleakage assessment. Our results regarding the superiority of the MTA-based sealer to the AH Plus® may be attributed to the differences between the components and properties of these two sealers. Endodontic sealers bond to dentin and gutta-percha to provide an optimal seal and to decrease the clinical microleakage [1]. The MTA-based sealer is hydrophilic and contains tricalcium silicate. It can set in wet environments. However, the AH Plus® sealer is hydrophobic, and moisture can adversely affect its sealing properties and bonding to the radicular dentin [21].

Based on our results, the apical seal in the groups irradiated with the diode laser was superior to that in the groups irradiated with the Nd:YAG laser, irrespective of the type of sealer, and the apical seal after the use of the Nd:YAG laser was superior to that in the groups that had not been irradiated with lasers. Thus, laser irradiation enhanced the apical seal, although this enhancement was not statistically significant. These results were similar to those of the study by Todea et al [1] in 2010. They also showed a lower level of apical microleakage in the laser-irradiated groups compared to the non-laser-treated groups; however, they showed similar levels of apical microleakage in the Nd:YAG-laser-irradiated and diode-laser-irradiated groups. The difference in this regard between their results and ours may be attributed to the method of microleakage assessment since they used En face OCT to measure the gap between the root canal filling material and radicular dentinal walls [1]. The difference between the laser-irradiated groups and the non-laser-irradiated groups may be explained by the fact that laser irradiation completely or partially removes the smear layer, while a thicker smear layer is present when the laser is not used, which interferes with sealer penetration and adhesion to dentinal tubules [2]; therefore, microleakage increases due to a poor adaptation. Laser irradiation also changes the morphology of the dentin by melting and resolidification as well as by formation of a glass-like surface with fewer defects compared to that in the non-laser-irradiated groups. This can affect the adaptation of the sealer to dentinal walls [1].

According to our results, irrespective of the type of sealer, the apical seal after the use of the diode laser was superior to that in the Nd:YAG-laser-irradiated groups, although the difference was not statistically significant. A lower apical seal in the Nd:YAG-laser-irradiated groups may be due to the fact that the Nd:YAG laser has a higher wavelength, and thus, it creates greater structural changes compared to the diode laser [8]. The Nd:YAG laser completely seals the dentinal tubules, while the diode laser relatively occludes the tubules. Due to the obstruction of dentinal tubules, the sealer cannot penetrate into the tubules, and therefore, a greater apical microleakage occurs. After the preparation of the root canals with the Nd:YAG laser, no difference was noted in the apical seal between the MTA-based and AH Plus® sealers. This finding may be attributed to the fact that in the Nd:YAG-laser-irradiated group, the dentinal tubules were completely sealed, and neither the MTA-based sealer nor the AH Plus® sealer could penetrate into the tubules. No similar study was found in this regard to compare our results with.

In the diode-laser-irradiated group, the apical microleakage was higher after the use of the MTA-based sealer in comparison to the AH Plus® sealer, but the difference was not significant. The reason for a lower apical microleakage after the use of the AH Plus® sealer was that the diode laser changes the smear layer and results in its evaporation. It also melts the radicular dentin [25]. These ultrastructural changes affect the hydrophobic AH Plus® sealer more than the hydrophilic MTA-based sealer. No similar study was available in this regard to compare our results with.

With regard to the time of occurrence of microleakage, the latest occurrence of microleakage was noted after the use of the Nd:YAG laser in combination with the MTA-based sealer, while the earliest occurrence of microleakage was noted after the use of the diode laser and the MTA-based sealer. These differences may be due to the changes caused in the dentin morphology by lasers and to the antibacterial effects of the sealer on the E.faecalis [26,27]. The SEM assessments showed that the diode laser caused a partial occlusion, whereas the Nd:YAG laser caused a complete obstruction of dentinal tubules. Some smear layer remained on the dentinal surfaces after the use of the diode laser; however, the Nd:YAG laser resulted in no residual smear layer on the surfaces. These findings were in agreement with those of the studies by Lopes et al [15] in 2016 and Moura-Netto et al [3] in 2012. Our results were slightly different from those of the study by Depraet et al [12] in 2005. They found that the Nd:YAG laser created open dentinal tubules and spherical particles due to a melted and fused smear layer that formed a glass-like structure [12].

In our study, the penetration of the sealer into the dentinal tubules was only noted after the use of the diode laser in combination with the AH Plus® sealer. In 2007, de Moura-Netto et al [8] showed that the penetration of the AH Plus® sealer into the dentinal tubules in the Nd:YAG-laser-irradiated group was greater than that after the use of the diode laser and also greater than that in the non-laser-irradiated groups. Such variability in the results may be due to the use of different parameters in the two studies such as different laser wavelengths and laser powers. In our study, both diode and Nd:YAG lasers resulted in a perfect adaptation of the AH Plus® sealer to the radicular dentin and a decreased adaptation of the MTA-based sealer to the radicular dentin. In general, it may be concluded that the application of diode laser, as an adjunct to root canal preparation, combined with the use of the AH Plus® sealer, has the best effect on the adaptation and penetration of the sealer into the radicular dentinal tubules.

Since most root canals are curved, future studies are required on the microleakage of sealers following the laser irradiation of curved root canals. Moreover, the present study had an invitro design, and therefore, a complete simulation of the clinical setting was not possible; thus, clinical trials are required to obtain more reliable results.

## CONCLUSION

Based on the results of this in-vitro study, diode and Nd:YAG laser irradiation, as an adjunct to root canal preparation, can decrease apical bacterial microleakage and dentin permeability, irrespective of the type of endodontic sealer. Application of diode laser, as an adjunct to root canal preparation, combined with obturation with the AH Plus® sealer would provide the best apical seal, although the differences found in our study were not statistically significant.
